# Seasonal Differences in Animal Welfare Assessment of Family Farming Dual-Purpose Cattle Raised under Tropical Conditions

**DOI:** 10.3390/ani8070125

**Published:** 2018-07-21

**Authors:** Adalinda Hernandez, Charlotte Berg, Rebecka Westin, Carlos Galina

**Affiliations:** 1 Department of Animal Environment and Health, Swedish University of Agricultural Sciences, SE 532 23 Skara, Sweden; Lotta.Berg@slu.se (C.B.); rebecka.westin@slu.se (R.W.); 2 Departamento de Reproducción, Facultad de Medicina Veterinaria y Zootecnia, Universidad Nacional Autónoma de México, 04510 México, D.F., Mexico; cgalina@unam.mx

**Keywords:** welfare assessment, tropical cattle, dry season, rainy season

## Abstract

**Simple Summary:**

Family-run cattle farms in the tropics deal with two distinct seasons, the dry and the rainy, where features such as resources, diseases and climate are variable. Nevertheless, an acceptable level of animal welfare must be maintained throughout the year. The purpose of this study was to investigate whether there were animal welfare issues at farms affected by either the dry or the rainy season. Forty-five dual-purpose family farms in the Mexican tropics were assessed via the Welfare Quality^®^ protocol. The animal welfare assessment on these farms obtained better results during the dry season, hence the season presenting a greater risk to animal welfare of dual-purpose cattle raised under tropical conditions is the rainy season. However, there were management-related differences observed between the two seasons, and the dry season also had some animal welfare threats. The fact that farms scored higher during the dry season is possibly the result of farmer awareness, leading to modification of their systems to provide animals with the necessary inputs to meet their production needs. If these modifications, such as providing supplementary feed and water points were not fulfilled, then welfare conditions might have been jeopardized.

**Abstract:**

Conditions on farms in the tropics can differ greatly depending on the season of the year. Characteristics such as disease prevalence, climate and availability of resources may not be constant all year around; however an acceptable level of animal welfare must be maintained throughout the year. Since it is neither practical nor economically feasible to perform several assessments per year, the purpose of this study was to define whether there were animal welfare issues at farms that were affected by the season to identify which season would present a greater risk to animal welfare, using a risk-based approach. Forty-five dual-purpose family farms in the Mexican tropics were assessed via the Welfare Quality^®^ protocol. During the rainy season, 2.2% of the farms were classified as excellent, 57.8% as enhanced, 31.1% as acceptable and 8.9% as unclassified. In the dry season, 31.1% were classified as excellent, 68.9% as enhanced and none of the farms were categorized as acceptable or unclassified. Consequently, the season which presented the greatest risk to animal welfare of dual-purpose cattle raised under tropical conditions was the rainy season. However, there were management-related differences observed between the two seasons and the dry season also had some animal welfare threats. The fact that farms scored higher during the dry season is possibly the result of farmer awareness, leading to modification of their systems to provide animals with the necessary inputs to meet their production needs. If these modifications were not fulfilled, then welfare conditions might have been jeopardized.

## 1. Introduction

Over the past few decades, consumer concerns about animal welfare have increased greatly, creating the need for standardized tools to assess animal welfare on farm animals (e.g., References [1,2]). However, most of these methods were created for intensive systems in industrialized countries [3], leading to the lack of standardized and scientifically proven protocols to assess the welfare of animals raised in tropical conditions where extensive family farming is predominant. In fact, family farming constitutes the main source of income for a large part of the population in these regions [4]. Furthermore, although the temperature in tropical regions may appear relatively stable year-round, there is still seasonal variation, for example in relation to the amount of precipitation and wind force, marking two distinct seasons: dry and rainy. Therefore, variations in the welfare of domestic animals can be anticipated.

The Welfare Quality^®^ (WQ^®^) protocol is one of the methods used to assess the overall level of welfare of farm animals. Developed as part of a project funded by the European Union (EU) in 2004, this method has become an important tool (e.g., References [5,6,7]). The method consists of animal-based on-farm welfare assessment protocols designed for intensive farms. There have been several attempts to adapt the WQ^®^ protocols to extensive production conditions (e.g., Reference [8]), including dual purpose family farming systems in the tropics [3]. Although some shortcomings have been identified, the protocols were still useful in assessing general animal welfare in these types of farms as well.

Conditions on farms in the tropics can differ greatly depending on the season of the year, where clear differences have been observed between the dry and rainy season. Characteristics such as disease prevalence, climate and availability of resources (water, feed and others) may not be consistent all year-round. However, an acceptable level of animal welfare should be maintained throughout the year. Since it is neither practical nor economically feasible to perform several assessments during the year, there is a need to identify the proper time to assess animal welfare in farms under tropical conditions in a reasonably representative way.

The purpose of the present study was to ascertain whether there are indicators related to animal welfare evaluated by the Welfare Quality^®^ protocol that are affected by either the dry or the rainy season, facilitating the choice of season from a risk-based perspective. 

## 2. Material and Methods

The study was carried out by evaluating 45 dual-purpose family farms at two different locations in Mexico. Twenty-three of the evaluated farms were in the municipality of Villa Corzo, Chiapas at 15°47′ N and 92°29′ W. The climate in this region is hot and sub-humid, with summer rainfall and average annual precipitation of 1247 mm. The remaining 22 farms were situated in the municipality of Tuxpan, Veracruz at 20°57′ N and 97°24′ W, which has a tropical climate with average summer rainfall of 996 mm. The studies were carried out during one dry and one wet season. The farms were selected based on willingness to participate, and farms covered by this study represented approximately 80% of the cattle farms in the relevant locations. According to colleagues with local knowledge, participating farms were not categorically different from those that chose not to participate. 

The farms were mainly focused on milk production, only male calves were sold for finishing, and after their last production cycle old cows were slaughtered for beef production. The herd size ranged from 7 to 90 cows and the size of the farms ranged from 4 to 15 hectares. Herds were mostly composed of crossbred animals *Bos taurus* and *Bos indicus*. The main breeds used for the crosses were Holstein, Zebu, Brown Swiss, Sardo Negro, Gyr and Jersey. 

The farms worked under a year-round, full time pasture system, and the animals were only gathered once per day during the mornings for milking which took a maximum of two hours per day. When gathered, the cows were given a supplementary feed consisting of chicken manure, ground corn and dry grass where the amount of supplementary feed was higher during the dry season. After milking, the cows were released to pasture on native grass species, such as *Hyparrhenia rufa, Digitaria decumbens*, *Panicum maximum*, *Cynodon nlemfluensis*, *Brachiaria brizanta*, *Brachiaria dictyoneura*, *Centrosema pubescens*, *Arachis pintoi*, *Clitoria ternatea*, *Canavalia ensiformis* and *Centrosema plumieri*. The cows stayed on the pasture unsupervised for the rest of the day and night, and the next morning they were returned to the milking parlor. Calves stayed together with the cows during the day and night, as did one or two bulls.

The average milk production per cow ranged from 8 to 16 L per day during the rainy season and from 7 to 12 L during the dry season. The cows were kept in the farms for as long as they were productive, approximately six lactation periods on average. Livestock facilities differed considerably between farms, from non-existent milking parlors or very rudimentary ones with just a roof made of wood and wire with capacity for one cow at a time, to concrete constructions with five to ten individual stalls equipped with a milking machine. Deworming and vaccination against brucellosis, leptospirosis, rhinotracheitis, bovine viral diarrhea and parainfluenza three, were routinely applied according to the national programme for disease control. According to the farmers, the farms did not have a herd veterinarian nor regular health checks, and they only called a veterinarian when a problem arose. Most farms did not have special facilities to quarantine sick animals; however, when an animal was sick, it was a common practice to separate it from the herd and supply medical treatment. No special measures were taken to prevent attacks from predators and other wild animals that could harm or stress the herds, such as cougars, jaguars, rattlesnakes, coyotes and other small felids which were present in the area. However, none of the farms reported any attacks.

### 2.1. Welfare Assessment

The farms were assessed once during the rainy season (July 2016) and once more during the dry season (January 2017). No special recommendations were made to improve or change, the welfare conditions of the animals between the two assessments. The assessments were performed by the same trained observer for each farm and area, using the Welfare Quality (WQ^®^) protocols for dairy and beef cattle [9] in accordance to Reference [3] (Table 1). This protocol was originally developed as part of the Welfare Quality project with a focus on housed cattle in temperate regions, and has since been used in several scientific studies on cattle welfare. Previous evaluations of the virtues and shortcomings of the protocol in relation to cattle raised under tropical conditions found it to be feasible [3]. 

The assessments were carried out via continuous observation of the animals for a period of 120 min on pasture. However, due to prevailing conditions on the farms in this study, some features were evaluated during the milking sessions when the animals were gathered in the milking parlor, where it was possible to perform the observations at an individual level. These observations covered the whole herd including cows, calves and bulls present. 

The generally small size of herds in the study meant that all the animals were observed and no sub-sampling was performed.

### 2.2. Calculation of Scores and Statistical Analysis

Calculations of scores for each criterion and welfare principle were performed according to the calculation model included in the WQ^®^ protocol [9]. The final results for each criterion and each principle were represented by a number from 0 to 100. Finally, the farms were classified according to four categories based on the final score in each principle, as follows: excellent was 80.1–100; improved was 60.1–80; acceptable was 20.1–60; and unclassified was 0–20.

Statistical analyses were carried out using Stata 14 (StataCorp LLC, College Station, TX, USA). For comparison between seasons, the non-parametric Wilcoxon-test for matched-pairs was used because the assumption of normality was often not met. Comparison between seasons was made at all levels (i.e., at each separate measure, criterion, principle and overall classification). 

## 3. Results

### 3.1. Overall Assessment of Animal Welfare

Results of the overall classification of farms are presented in Table 2.

During the rainy season, 4 out of the 45 farms did not achieve the minimum score to be classified as acceptable, 14 were classified as acceptable, 26 as enhanced and one as excellent. During the dry season, 31 of the farms were classified as enhanced, fourteen as excellent, and no farms were placed in the acceptable or unclassified categories. It was noted that, animal welfare problems were more prominent during the rainy season.

### 3.2. Separate Measures

At the level of separate measures, the percentages of lean cows, cows covered in dirt, integument alterations, lameness, nasal discharge, ocular discharge, number of coughs, hampered respiration, diarrhea, mortality and frequency of agonistic behaviors were higher in the rainy season compared to the dry season. However, the qualitative behavior assessment scores were lower and the good human-animal relationships were better in the rainy than in the dry season. The mean and range for each measure and season are displayed in Table 3.

### 3.3. Animal Welfare Criteria and Principles

The average scores for the four animal welfare principles evaluated (good feeding, good housing, good health and appropriate behavior), are presented in Table 4. In general, the highest scores were obtained for good housing and appropriate behavior and good feeding received the lowest scores. 

Scores for all four principles were lower during the rainy season compared to the dry season. Regarding good feeding, both absence of prolonged hunger and absence of prolonged thirst scored lower in the rainy season, but the differences between the seasons were larger for absence of prolonged hunger. Comfort around resting was also lower during the rainy season, and ease of movement did not change, which was not surprising as the animals were on pasture all day all year-round. Absence of injuries and absence of disease scored higher during the dry season, whereas absence of pain induced by management procedures remained the same regardless of season. The scores for expression of social behaviors were higher during the dry season and those for good human-animal relationship and positive emotional state were higher during the rainy season.

### 3.4. Water Sources

**Figure 1:**
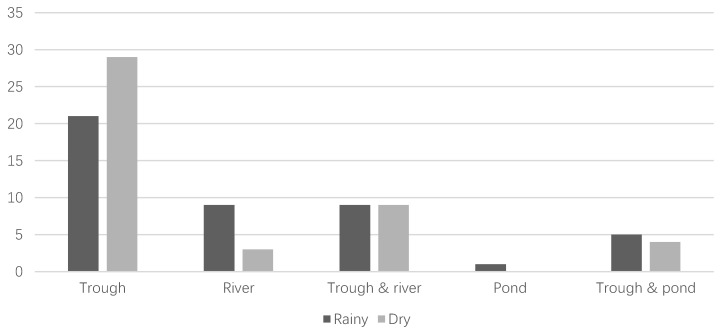
Assessment of sources of drinking water for the animals in this study (*n* = 45) during the two seasons. Three different sources of water were observed: troughs, rivers and ponds, as well as two different combinations i.e., trough and river and trough and pond.

The animals at the farms had three different sources of drinking water, i.e., troughs: artificial containers intended to provide water to animals, rivers and natural flowing watercourses, and ponds: natural or artificial pits in the ground. Some farms utilized two different sources simultaneously. In Figure 1, it can be observed that water sources varied between the two seasons. Troughs were the most common source of water supply in both the rainy and dry season, with 21 farms exclusively using troughs during the rainy season and 29 during the dry. In the rainy season, nine farms used rivers, whereas only three farms used rivers as the only source of water during the dry season. The combination of troughs and rivers was equally present where nine farms used them during both seasons. Ponds were also used during the rainy season where one farm used a pond as the main source of water and five farms used ponds in combination with troughs. In the dry season only four farms used the combination of troughs and ponds. 

## 4. Discussion

The aim of this study was to identify which season constituted a higher risk to animal welfare in dual-purpose cattle raised under extensive tropical conditions. It is important to identify the higher-risk season, if any, when welfare assessments are more likely to identify welfare problems. Using the Welfare Quality^®^ assessment protocol, it was found that farms scored higher, i.e., had better animal welfare, during the dry season as opposed to the rainy season. This applied to both the principle and most features at the criteria level. 

During the dry season, when the availability of food at pasture was lower [10], the animals often received supplementary feeding in higher quantities. Additionally, most tropical grasses were not sufficient in nitrogen content [8]; thus, a larger portion of supplementary feeding fulfilled this nutritional need resulting in better body conditions for the cattle. The fact that more animals were leaner during the rainy season implied supplementary feeding was probably needed during the rainy season as well. It should be kept in mind that seasons are not static; the situation in relation to pasture nutritional content can be quite different when comparing the beginning of the dry season to the end of the dry period, and the same applies for the rainy season. Hence, it may be necessary to use extra supplementary feed at the end of the dry season due to the poor quality of pasture grass at that time of the year. This together with the stress of calving [11], could compromise animal welfare.

Most of the natural sources of water in the two areas where the farms were situated, depended directly on the amount of rain. During the rainy season these sources seemed to be larger and more frequently observed at the farms than during the dry season. However, when there was no natural source of water available for the animals, there were troughs allocated on the pasture area. These were usually cleaner and easier to measure under the standards of the Welfare Quality^®^ protocol, and resulted in higher scores. Furthermore, the need for water intake differed between the two seasons since it is also related to dry matter intake [12,13,14]. *Bos indicus* cattle needed around 10 L of water/kg of dry mater consumed [15], therefore the need for good water sources was more relevant during the dry season. 

Comfort around resting scored lower during the rainy season. This was attributed to the average time to it took to lie down, plus the muddy slippery surfaces created by rain during this period. Lying down is important for cattle because this is when the animals rest and ruminate [16]. However, the differences were marginal and did not seem to prevent the cows from lying down. 

Under the good health principle, the overall scores for absence of injuries were higher during the dry season, and all farms were considered as acceptable during both seasons. Using the criterion of absence of disease, the farms scored lower during the rainy season, and during the dry season all farms scored the minimum to be classified as acceptable. However, during the rainy season there were farms which failed to achieve the minimum scores to be placed in this category. The transmission of pathogens can be influenced by different factors, such as population density and resources distribution, among others [17]. Due to the availability and distribution of pastures during the dry season, it was observed that animals would spread looking for good sources of grass, making their interactions scarce.

The principle of appropriate behavior did not differ between the seasons. This result was expected, as the cows were kept outdoors at pasture all year-round and space allowance was more generous. However, under the criterion of good human-animal relationship and positive emotional state, the farms scored higher during the rainy season; this could be due to the availability of resources, and pastures were more abundant and nutritive during the rainy season whereas more nutritional stress occurred during the dry season [10]. In addition, as the rainy season was the calving period, this influenced the behavior of cows towards humans as well as their emotional state. This is without ruling out the possibility of stress due to presence of predators, which some studies have shown to be more abundant during the dry season [18,19,20].

According to the results in this study, the season with the greatest risk to animal welfare of dual-purpose cattle raised under tropical conditions was the rainy season. Therefore, it could be argued this is the most appropriate season to conduct animal welfare inspections. However, it is important to mention that the dry season also presented certain risks to the animals as they were constantly kept at pasture. The reason farms in this study scored higher during the dry season is possibly the result of farmers being aware of the risks related to feed and water shortage during this period; thus, they modified their systems to provide the animals with the necessary inputs to meet their production needs. If these modifications, such as providing supplementary feed and additional water points are not fulfilled the welfare conditions could greatly deteriorate. Hence, it may still be necessary to perform welfare inspections during the dry season to ensure a proper level of welfare for the animals all year-round. It is important to consider the variations within each season since the characteristics were not the same in the beginning as at the end of each season. It could be useful and important to identify if there is a marked difference between these variations to have a more accurate assessment of animal welfare. This study was carried out during one dry and one wet season only. There was generally very little between-year variation in the manifestation of the seasonal climate variation in this region. Nevertheless, repeating the study over several years may be beneficial.

## 5. Conclusions

We conclude that overall animal welfare can be more at risk during the rainy season, however if certain management modifications are not performed during the dry season, the welfare conditions might also be jeopardized during this period.

## Figures and Tables

**Table 1:** Principles, criteria and measures of the Welfare Quality^®^ protocol used for the assessment. (Only a description of the adaptations for these tropical dual-purpose farms is provided below, the full protocol can be found at Reference [9]).

Principles	Criteria	Measures
Good feeding	Absence of prolonged hunger	Body Condition Score (% of very lean animals)
	Absence of prolonged thirst	Availability and cleanliness of water: Including natural water sources (rivers, streams and ponds). No presence of foul odors and/or abnormal colors, still or running water.
Good housing	Comfort around resting	Duration of lying down. % collisions with housing equipment, this event was only possible to assess at the milking parlor as it was the only area with housing equipment. Lying outside lying area (dry and clean area which did not lead to any trauma with objects and was covered by natural or artificial shade) Cleanliness, % of dirty animals.
	Ease of movement	Not applicable in these farm systems.
Good health	Absence of injuries	Lameness. Integument alterations.
	Absence of disease	Number of coughs, nasal and ocular discharge, impaired breathing, diarrhea, bloated rumen, mortality.
	Absence of pain induced by management procedures	Dehorning, disbudding, castration and tail-docking. Use of anesthetics or/and analgesics for the mentioned procedures.
Appropriate behavior	Expression of social behavior	Agonistic and cohesive interactions
	Expression of other behaviors	Access to pasture. In accordance with the prevailing conditions of these farms, animals spent the whole day at pasture, with the exception of milking time, which took no longer than two hours per day.
	Good human-animal relationship	Avoidance distance, recorded during milking, in an open but limited space, where the animals had the chance to avoid the evaluator touch if desired.
	Positive emotional state	Qualitative behavioral assessment.

**Table 2:** Number and percentage of farms according to their final classification of assessment with the Welfare Quality protocol. *n* = 45.

Classification of Animal Welfare	Rainy Season		Dry Season	
	No. of Farms	Percentage	No. of Farms	Percentage
Excellent	1	2.2	14	31.1
Enhanced	26	57.8	31	68.9
Acceptable	14	31.1	0	0
Unclassified	4	8.9	0	0

**Table 3:** Results of separate measures of the Welfare Quality^®^ assessment on 45 farms during the rainy and dry season.

Criteria	Measures	Rainy Season			Dry Season			*p*-Value
		Mean	Min	Max	Mean	Min	Max	
Absence of prolonged hunger	% lean cows	28.9	0	80	4.7	0	20	<0.001
Comfort around resting	Mean time to lie down (s)	3.5	2	5.4	2.8	2	4.3	0.48
	% cows lying outside the lying area	0	0	0	0	0	0	-
	% dirty cows	7.6	0	86.0	2.7	0	20.0	0.08
Absence of injuries	% mild integument alterations	8.3	0	73.0	7.5	0	46.7	0.17
	% severe integument alterations	4.8	0	33.0	1.0	0	20.0	0.29
	% moderate lameness	3.0	0	13.0	0.5	0	13.3	0.002
	% severe lameness	0	0	0	0	0	0	-
Absence of disease	% nasal discharge	7.9	0	33.0	1.8	0	20.0	<0.001
	% ocular discharge	6.5	0	33.0	2.7	0	26.7	0.004
	No. coughs/15 min	1.7	0	9.0	0.5	0	4.0	0.04
	% hampered respiration	1.4	0	33.0	0.7	0	13.0	0.96
	% bloated rumen	0	0	0	0	0	0	-
	% diarrhea	3.4		27.0	2.6	0	20.2	0.98
	% mortality	3.3	0	21.0	1.8	0	14.0	0.62
Expression of social behavior	Frequency of agonistic behaviors	1.4	0	6.0	0.3	0	0.9	0.001
	Frequency of cohesive behaviors	0.5	0	3.0	1.4	0	5.0	0.001
Good human-animal relationship	% cows that could be touched	83.1	6	100	72.0	0	100	0.001
	% cows that could be approached by 50 cm	13.7		87.0	23.0	0	100	0.001
	% cows that could be approached by 100 cm	2.9	0	30.0	4.3	0	26.7	0.04
	% cows that could not be approached	0.3	0	9.5	0.7	0	10.0	0.64
Positive emotional state	QBA * index	4.2	−2.5	6.0	5.2	−1.9	6.0	<0.001

* Qualitative behavioral assessment (QBA).

**Table 4:** Results of calculated scores for animal welfare principle and criteria levels in the Welfare Quality assessment on 45 farms during the rainy and dry season, respectively. Principle level scores highlighted in bold.

Principle	Criteria	Rainy Season			Dry Season			*p*-Value
		Mean	Min	Max	Mean	Min	Max	
**Good feeding**		**28.6**	**0.2**	**100**	**59.4**	**30.8**	**100**	**<0.001**
	Absence of prolonged hunger	32.6	3.8	100	71.8	30.3	100	<0.001
	Absence of prolonged thirst	57.7	0	100	73.1	29.0	100	0.02
**Good housing**		**89.5**	**40.4**	**100**	**96.0**	**72.1**	**100**	**0.008**
	Comfort around resting	86.5	23.6	100	94.9	64.2	100	0.003
	Ease of movement ^1^	100	100	100	100	100	100	-
**Good health**		**48.3**	**9.6**	**100**	**68.7**	**18.4**	**100**	**<0.001**
	Absence of injuries	88.0	43.9	99.7	96.5	61.6	100	0.002
	Absence of disease	52.5	7.3	100	75.5	22.3	100	0.28
	Absence of pain induced by management procedures ^2^	29.5	15.7	100	29.5	15.7	100	-
**Appropriate behavior**		**82.2**	**26.0**	**96.1**	**89.1**	**58.7**	**100**	**0.02**
	Expression of social behavior	81.3	19.0	100	87.2	65.7	100	0.007
	Expression of other behaviors ^1^	100	100	100	100	100	100	-
	Good human-animal relationship	93.8	65.4	100	89.6	72.3	100	<0.001
	Positive emotional state	94.8	25.8	100	85.9	17.2	100	<0.001

^1^ Kept at pasture during the whole year. ^2^ Management of painful procedures did not differ between assessments.
